# Ocular Manifestations in Psoriasis Screening (OcMaPS) Questionnaire: A Useful Tool to Reveal Misdiagnosed Ocular Involvement in Psoriasis

**DOI:** 10.3390/jcm10051031

**Published:** 2021-03-03

**Authors:** Angelo Ruggiero, Gabriella Fabbrocini, Sara Cacciapuoti, Eleonora Cinelli, Lucia Gallo, Matteo Megna

**Affiliations:** Section of Dermatology, Department of Clinical Medicine and Surgery, University of Naples Federico II, Via Pansini 5, 80131 Napoli, Italy; angeloruggiero1993@libero.it (A.R.); gafabbro@unina.it (G.F.); sara.cacciapuoti@libero.it (S.C.); elecinelli@gmail.com (E.C.); luciagallo890@gmail.com (L.G.)

**Keywords:** psoriasis comorbidity, ocular psoriasis, ocular involvement in psoriasis, screening questionnaire

## Abstract

Psoriasis is an immune-mediated, chronic inflammatory disease, which mainly affects the skin, although it has systemic pathological effects. Comorbidities of psoriasis include ocular disorders, which are often nonspecific or mildly symptomatic. The aim of this study was to show the importance of ocular-disease screening in psoriatic patients using the Ocular Manifestations in Psoriasis Screening (OcMaPS) questionnaire. Patients suffering from moderate-to-severe psoriasis referring at our outpatient-clinic were consecutively enrolled. Each patient was asked to complete a screening questionnaire (OcMaPS). Patients reporting ocular symptoms were referred for an ophthalmological examination. A total of 372 patients were enrolled in the study. Ocular symptoms were detected in 39 patients (10.5%), and 37 patients were referred to ophthalmological examination which confirmed the presence of ocular manifestation in 30 patients. There were three cases (10%) of uveitis, 14 (46.6%) of dry eye and 13 (43.3%) of cataract, in progress or already treated with surgery. In the remaining seven patients, no ocular manifestations were found. Ocular manifestations in psoriatic patients are not rare. It is important to be aware of ocular symptoms in psoriatic patients, screening patients (with a consultation or OcMaPS questionnaire), which leads to earlier diagnosis and treatment.

## 1. Introduction

Psoriasis is an immune-mediated, chronic inflammatory disease of genetic basis, which mainly affects the skin, although it has systemic pathological effects [1,2]. Indeed, psoriasis has been shown to be associated with several comorbidities, such as psoriatic arthritis (PsA), Crohn’s disease (CD), psychological/psychiatric disorders, and ocular diseases [3]. In particular, several ocular manifestations have been reported including uveitis, dry eye, retinal abnormalities, blepharitis, conjunctivitis, keratitis, iridocyclitis, UV-induced cataracts, and birdshot chorioretinitis [4,5]. In particular, patients with PsA show an elevated risk for developing uveitis, which is a well-known ophthalmologic manifestation of seronegative spondyloarthropathy [5,6]. Ocular manifestation prevalence in psoriasis patients without joint involvement seems less frequent than in PsA, with published studies reporting variable frequency data [7,8,9]. Moreover, psoriasis treatments may also lead to ocular complications, with a higher prevalence of ocular manifestations in patients treated with methotrexate, followed by acitretin and biologic drugs, and less frequent in cases treated with topical steroid, phototherapy, and cyclosporine [10]. It has been reported that there is a higher eye involvement prevalence in men than in women, and this is almost always preceded by skin manifestations. In former publications, reported incidence rates for ocular involvement in these patients varied from 10% to 58% [10,11,12,13]. Hence, ocular manifestations in psoriasis are not uncommon [7,10,14,15,16], with higher frequency reported among PsA and pustular psoriasis patients [4,14]. In addition, ocular manifestations are often nonspecific or mildly symptomatic, and not appreciated by both patients or physicians; hence ocular manifestations may be often misdiagnosed. Due to the systemic nature of psoriasis, the dermatologist should act as a sentinel physician, screening subjects for signs and symptoms which may require further evaluation, leading to a prompt diagnosis and treatment of psoriasis-related comorbidities. Hence, on the basis of the ocular manifestations in inflammatory bowel diseases (IBD) screening (OMIS) questionnaire [17], which investigated ocular symptoms in IBD patients, we developed the Ocular Manifestations in Psoriasis Screening (OcMaPS) questionnaire, administered by dermatologists to psoriatic patients in order to early discover ocular symptoms that may require an ophthalmologic examination.

## 2. Materials and Methods

Patients suffering from psoriasis admitted to the Psoriasis Care Unit (face-to-face visits or tele-dermatology service) were consecutively enrolled. Inclusion criteria were as follows: (i) diagnosis, by a dermatologist, of moderate-to-severe plaque psoriasis lasting at least one year; (ii) ability to understand and complete the questionnaire; and (iii) patient under conventional or biologic systemic treatments as well as small molecules. Exclusion criteria were as follows: (i) patient undergoing only topical treatments; (ii) patient with other autoimmune systemic comorbidities (lupus, sarcoidosis, etc.). After obtaining informed consent, each enrolled patient was asked to complete a screening questionnaire (OcMaPS), as well as privacy statements. The OcMaPS (Ocular Manifestations in in Psoriasis Screening) questionnaire (Table 1), was developed on the basis of the OMIS questionnaire, with the aim of collecting data about ocular symptoms among psoriatic patients, in order to select which patients needed an ophthalmologic evaluation as well as investigating on ocular manifestations frequency among psoriasis subjects. Patients were asked to complete the questionnaire during or at the end of the visit. Section A of the questionnaire collected the patient’s clinical and demographic characteristics, personal data, psoriasis data (psoriasis duration, PsA duration if applicable), comorbidities, and ongoing psoriasis treatments. Section B collected data about ocular manifestations and investigated (i) family history of ocular diseases, (ii) ongoing or previous therapy for ocular manifestations, (iii) previous diagnosis of eye diseases and the number of episodes per year, (iv) ocular symptoms presented during 3 months prior to completion of the questionnaire, (v) any need for eye drops in the last 3 months and response to treatment, and (vi) any complications due to a previous ocular inflammation. Patients reporting at least one ocular symptom as well as patients with a history of ocular manifestations were referred to an ophthalmologist who performed a complete ophthalmological examination.

Continuous variables were displayed as mean ± standard deviation, whereas categorical variables were displayed as a number and proportion of patients. The unpaired Student’s *t*-test was used to calculate the significance of differences in mean values at the different time points of treatment. A *p*-value of < 0.05 was considered statistically significant. All statistical analyses were performed using GraphPad-Prism 4.0 (GraphPad Software Inc., La Jolla, CA, USA).

## 3. Results

A total of 387 patients completed the questionnaire. However, 15 patients were excluded since they did not answer to all questionnaire items. Hence, a total of 372 patients (192 males (51.6%) and 180 females (48.4%)), with a mean age of 50.6 ± 16.6 years, were considered for the study. Mean psoriasis duration was 24.4 ± 10.8 years and 25% (*n* = 93) were also affected by PsA with a mean duration of 7 ± 5.3 years. The most frequent comorbidity was hypertension (33.6%, *n* = 125), followed by dyslipidaemia (23.6%, *n* = 88), cardiopathy (13.4%, *n* = 50), depression (10.4% *n* = 39), and diabetes (5.6%, *n* = 21). Ongoing traditional systemic psoriasis treatments included methotrexate (9.9%, *n* = 37), cyclosporine (5.4%, *n* = 20), acitretin (5.9%, *n* = 22), and narrow band UVB phototherapy (5%, *n* = 18). Ongoing biologic treatments included adalimumab (13.7%, *n* = 51), ixekizumab (13.4%, *n* = 50), ustekinumab (12.6%, *n* = 47), secukinumab (11.3%, *n* = 42), apremilast (6.7%, *n* = 25), guselkumab (5.3%, *n* = 20), etanercept (4.3%, *n* = 16), risankizumab (3.5%, *n* = 13), certolizumab (3.2%, *n* = 12), and infliximab (0.2%, *n* = 1). Eighty-two (22.9%) patients had a positive family history of ocular diseases (cataracts (12.6%, *n* = 47), glaucoma (6.7%, *n* = 25), retinal detachment (1.6%, *n* = 6), and uveitis (1.1%, *n* = 4)). Ocular symptoms were detected in 39 patients (10.5%). Among these, 37 patients were referred to an ophthalmologist (20 males (55.5%) and 17 females (44.5%) with a mean age of 48.6 ± 16.8 years). The other two patients refused ophthalmologic examination. Among these 37 patients, the most frequent eye-symptoms, reported either individually or in association with each other, were red eye (46.6%, *n* = 17), ocular pain (33.3%, *n* = 12), photophobia (7.4%, *n* = 9), and visual fogging (8.9%, *n* = 3). Ophthalmological examination confirmed the presence of ocular manifestation in 30 patients. There were three cases (10%) of uveitis (two of them had a personal medical history of uveitis, one patient was diagnosed for the first time), 14 cases (46.6%) of dry eye, and 13 cases (43.3%) of cataract, in progress or already treated with surgery. In these 30 patients, the most frequent treatments were adalimumab (20 %, *n* = 6) and acitretin (20%, *n* = 6), followed by etanercept (16.6%, *n* = 5), methotrexate (13.3%, *n* = 4), ixekizumab (10%, *n* = 3), secukinumab (6.6%, *n* = 2), narrow-band phototherapy (6.6%, *n* = 2), ustekinumab (3.3%, *n* = 1), and apremilast (3.3%, *n* = 1). In the other seven patients who reported visual fogging and red eye in their questionnaires, and who were evaluated by ophthalmologists, no ocular manifestations were found. Among patients with confirmed ocular manifestations the mean psoriasis duration was significantly higher with respect to patients without ocular complaints (27.2 ± 6.2 vs. 22.4 ± 8.5; *p* < 0.01).

## 4. Discussion

The chronic inflammation that sustains psoriasis has been related to higher levels of pro-inflammatory cytokines [18,19,20]. Indeed, it has been shown that the central role is played by the activation of the tumor necrosis factor (TNF)-α pathway as well as increased inflammasome activity and interleukin (IL)-17 and IL-23 [18,19,20,21]. It is now well-established that patients with severe psoriasis have an excess of systemic inflammation, with a related higher mortality if compared to the general population [22]. It is believed that psoriasis is not merely a skin disease but other organs like eyes can be affected by a chronic systemic inflammation. However, to date only few studies have evaluated ocular manifestations in psoriasis [7,8,9,13,19]. Eye involvement in psoriasis may be underestimated by dermatologists, although psoriasis has been acknowledged as a systemic disease for decades. Ophthalmic complications of psoriasis are numerous and can affect almost any part of the eye [23]. Indeed, people with severe psoriasis and those with mild psoriasis have an increased risk of uveitis [15,24]. Increasing evidence supports Th17 cells as key mediators of ocular inflammatory disease, such as uveitis [25]. In term of pathogenesis, both psoriasis and uveitis are considered as paradigms of T-helper 1/T-helper 17 (Th1/Th17) inflammatory reactions [26]. Certain cytokines such as TNF-α, IL-17, IL-23, and IL-6 play a significant role in the pathogenesis of both psoriasis and uveitis. The partial common inflammatory scenario behind these two diseases could explain the increased incidence of uveitis in psoriatic patients (reported incidence estimated to occur in 7–20% of the psoriasis cases) [27]. On the other hand, psoriasis treatments may also lead to ocular complications [12]. Interestingly, a case–control study evaluating 200 psoriatic patients and 100 healthy controls showed that ocular findings in patients under treatment were associated with the type of treatment and more frequent in those treated with methotrexate, followed by acitretin and biologic drugs, and less frequent in cases treated with topical steroid, phototherapy, and cyclosporine [13]. Indeed, acitretin treatment may be commonly related with drying and inflammation of mucous membranes (e.g., conjunctivitis and xerophthalmia) and rarely with vision blurred, night blindness, or ulcerative keratitis [28]. During narrow-band-UVB treatment sessions, because of the risk of induction of cataract, ocular protection is mandatory. However, in the absence of symptoms or a known ocular disorder, prior ophthalmologic control is not considered necessary [28]. As regards biologics, a recent review highlighted a variety of data associating the new onset of uveitis, as a paradoxical effect of anti-TNF therapy in rheumatic conditions, predominantly under etanercept [29]. On the other hand, adalimumab has been approved in uveitis and inflammatory bowel diseases, indirectly showing a common inflammatory background in these diseases [15]. An early recognition of ocular manifestation and a strict control of inflammatory activity in uveitis as well as in other ocular diseases may led to a dramatic reduction in the incidence of vision-threatening secondary complications [28,29]. However, the association between various ocular disorders and psoriasis is likely to be missed in patients if the physician is not specifically aware. For these reasons, physicians should maintain a high index of suspicion that ophthalmic symptoms in patients with psoriasis may be related to their underlying disease, even though signs and symptoms are often vague. Indeed, ocular complaints are often non-specific or mildly symptomatic, so the clinical relevance may not be appreciated by the patient or the physician taking care of psoriasis patients and, therefore, may be misdiagnosed. However, to date, ophthalmologic screening or evaluation is still not recommended in official recommendations and guidelines into the already-large academic framework of psoriasis research and care [30]. Our study showed a positive personal history of ongoing ocular manifestations in 39 patients (10.5%). Among these, two refused ophthalmologic consultation, while 37 patients were referred to a specialist ophthalmologic consultation. Ophthalmological history and visits confirmed the presence of ocular manifestation, both past and active, in 30 patients. There were three cases (10%) of uveitis (two of them had a personal medical history of uveitis, one patient was diagnosed for the first time), 14 cases (46.6%) of dry eye, and 13 cases (43.3%) of cataract, in progress or already treated with surgery. In these 30 patients, the most frequent treatments were adalimumab (20 %, *n* = 6) and acitretin (20%, *n* = 6), followed by etanercept (16.6%, *n* = 5), methotrexate (13.3%, *n* = 4), ixekizumab (10%, *n* = 3), secukinumab (6.6%, *n* = 2), narrow-band phototherapy (6.6%, *n* = 2), ustekinumab (3.3%, *n* = 1), and apremilast (3.3%, *n* = 1). In the remaining seven patients, no ocular manifestations were found; they complained of red eye or visual fogging when completing the questionnaire. Hence, our results show that ocular manifestations in psoriatic patients are not rare, highlighting the importance of ocular screening even if patients does not spontaneously report ocular manifestations. Our data are based on the OcMaPS questionnaire, a specific questionnaire proposed as a simple tool for ophthalmological screening in psoriatic patients in non-specialist settings. It may represent a qualitative screening tool completed by patients themselves during or at the end of visits, with the main purpose of detecting potential eye involvement in psoriatic patients. The need for an integrated multidisciplinary approach has recently received growing attention in psoriatic patients. However, published literature has focused mainly on articular, metabolic, gastrointestinal, and psychiatric aspects, for which dedicated screening questionnaires have been developed to be used for the early detection of systemic involvement. To the best of our knowledge, this is the first screening questionnaire investigating ocular symptoms among psoriatic patients. Moreover, our results show the importance of ocular screening in psoriatic patients, highlighting that mild symptoms, if not well detected, may be erroneously ignored by patients and misdiagnosed by physicians. Hence, it is important to look for ocular symptoms in psoriatic patients, using at least one ophthalmologic evaluation or a screening questionnaire in order to select patients needing a specialist consultation. Moreover, it should be mandatory to inform patients of potential eyes involvement, leading patients to be aware of this emergent association. However, more studies are needed to confirm our data, to understand the real incidence of ocular involvement in psoriasis, and to validate the proposed OcMaPS questionnaire as a useful tool to screen psoriatic patients in a non-specialist setting.

## 5. Limitation

The major limitation of this study is the lack of a control group. Moreover, 13.7% of our patients were treated with adalimumab which is also used in uveitis treatments. Hence, this may lead to a reduction in reported ocular symptoms. Another limitation of the study is that ocular manifestations may be undervalued and not appreciated by patients because they are mildly symptomatic. Therefore, it is possible, in clinical practice, that patients with psoriasis did not declare ocular complaints when requested through the OcMaPS questionnaire.

## Figures and Tables

**Table 1:** OcMaPS (Ocular Manifestations in in Psoriasis Screening) questionnaire.

Section A: Demographic Clinical and Disease Features	Section B: Eye Involvement
1. Surname and name 2. Birth date 3. Age 4. Sex: M/F 5. Comorbidities: (a) Hypertension (b) Dyslipidaemia (c) Depression (d) Cardiopathy (e) Other (please specify) 6. Psoriasis: YES/NO 7. Psoriasis duration (in years) 8. Psoriatic arthritis: YES/NO 9. Psoriatic arthritis duration time (in years) 10. Current treatment(s): (a) Phototherapy (b) Cyclosporine (c) Methotrexate (d) Acitretin (e)Etanercept (f) Adalimumab (g) Infliximab (h) Secukinumab (i) Ixekizumab (l) Brodalumab (m) Certolizumab (n) Ustekinumab (o) Guselkumab (p) Risankizumab (q) Tildrakizumab (r) Golimumab (s) Apremilast	1. Is any member of your family affected by any of the following eye diseases? (a) Uveitis (b) Glaucoma (c) Cataracts (d) Retinal detachment 2. Are you under therapy with ocular drugs? YES/NO 3. Which one/ones? 4. Have you ever been diagnosed with: (a) Dry eye (b) Uveitis (c) Scleritis (d) Episcleritis (e) Conjunctivitis (f) Other (ocular) 5. In the last 3 months have you suffered from: (a) Red eye, mono/bilateral (b) Eye pain (c) Photophobia (persistent discomfort to light) (d) Visual fogging or persistent visual impairment for at least 24 h, mono/bilateral 6. Have you practiced eye therapy with eye drops in the last 3 months? 7. In the case of a diagnosis ascertained by the ophthalmologist of uveitis/scleritis/episcleritis, how many episodes occur per year? (a) One to two (b) Three to six (c) More than six 8. Did the only treatment with eye drops resolve the symptoms? YES/NO 9. Has it been necessary to use oral or systemic drugs? YES/NO 10. Since the diagnosis of psoriasis, did you have ocular manifestations? YES/NO 11. Referral to the ophthalmologist: YES/NO

## Data Availability

The data that support the findings of this study are available from the corresponding author upon reasonable request.
